# Evaluation of a novel sodium borocaptate-containing unnatural amino acid as a boron delivery agent for neutron capture therapy of the F98 rat glioma

**DOI:** 10.1186/s13014-017-0765-4

**Published:** 2017-01-23

**Authors:** Gen Futamura, Shinji Kawabata, Naosuke Nonoguchi, Ryo Hiramatsu, Taichiro Toho, Hiroki Tanaka, Shin-Ichiro Masunaga, Yoshihide Hattori, Mitsunori Kirihata, Koji Ono, Toshihiko Kuroiwa, Shin-Ichi Miyatake

**Affiliations:** 1 Department of Neurosurgery, Osaka Medical College, 2-7 Daigakumachi, Takatuki-shi, Osaka, Japan; 2 Kyoto university research reactor institute, 2, Asahiro-Nishi, Kumatori-cho, Sennan-gun, Osaka Japan; 3 Reserch Organization for the 21th Century, Osaka Prefecture University, 1-1 Gakuen-cho, Nakaku, Sakai, Japan; 4 Division for Advanced Medical Development, Cancer Center, Osaka Medical College, 2-7 Daigakumachi, Takatuki-shi, Osaka, Japan

**Keywords:** Boron neutron capture therapy, F98 rat glioma model, ACBC-BSH

## Abstract

**Background:**

Boron neutron capture therapy (BNCT) is a unique particle radiation therapy based on the nuclear capture reactions in boron-10. We developed a novel boron-10 containing sodium borocaptate (BSH) derivative, 1-amino-3**-**fluorocyclobutane-1-carboxylic acid (ACBC)-BSH. ACBC is a tumor selective synthetic amino acid. The purpose of this study was to assess the biodistribution of ACBC-BSH and its therapeutic efficacy following Boron Neutron Capture Therapy (BNCT) of the F98 rat glioma.

**Methods:**

We evaluated the biodistribution of three boron-10 compounds, ACBC-BSH, BSH and boronophenylalanine (BPA), in vitro and in vivo, following intravenous (i.v.) administration and intratumoral (i.t.) convection-enhanced delivery (CED) in F98 rat glioma bearing rats. For BNCT studies, rats were stratified into five groups: untreated controls, neutron-irradiation controls, BNCT with BPA/i.v., BNCT with ACBC-BSH/CED, and BNCT concomitantly using BPA/i.v. and ACBC-BSH/CED.

**Results:**

In vitro, ACBC-BSH attained higher cellular uptake F98 rat glioma cells compared with BSH. In vivo biodistribution studies following i.v. administration and i.t. CED of ACBC-BSH attained significantly higher boron concentrations than that of BSH, but much lower than that of BPA. However, following convection enhanced delivery (CED), ACBC-BSH attained significantly higher tumor concentrations than BPA. The i.t. boron-10 concentrations were almost equal between the ACBC-BSH/CED group and BPA/i.v. group of rats. The tumor/brain boron-10 concentration ratio was higher with ACBC-BSH/CED than that of BPA/i.v. group. Based on these data, BNCT studies were carried out in F98 glioma bearing rats using BPA/i.v. and ACBC-BSH/CED as the delivery agents. The corresponding mean survival times were 37.4 ± 2.6d and 44.3 ± 8.0d, respectively, and although modest, these differences were statistically significant.

**Conclusions:**

Our findings suggest that further studies are warranted to evaluate ACBC-BSH/CED as a boron delivery agent.

## Background

Various radiation therapy modalities (e.g., stereotactic radiosurgery, intensity-modulated radiation therapy and particle radiation therapy) have been used in attempts to improve the prognosis of high-grade gliomas, but the results have hardly advanced over the past few decades, for the following two reasons. First, as high-grade gliomas are highly infiltrative to normal brain parenchyma, the tumor cells can migrate as far as several centimeters away from the periphery of the main tumor mass, and thus beam-limiting irradiation is insufficient to control microscopic tumor clusters in surrounding normal brain. The second reason is the low radiosensitivity of glioma cells. It is almost impossible to eliminate all of the infiltrating tumor cells within the limits of tolerance for normal brain. Thus, the selective destruction of tumor cells that have infiltrated normal brain is an even more difficult challenge compared with neoplasms at other organs.

At our institution, we have clinically applied boron neutron capture therapy (BNCT) since 2002 as an experimental adjuvant therapy for patients with recurrent or newly diagnosed malignant gliomas, and we have achieved superior outcomes compared with those of the standard treatment using fractionated photon irradiation [1, 2]. BNCT is a type of particle radiotherapy requiring low-energy thermal neutrons and non-radioactive boron-10 (^10^B) compounds that can selectively accumulate in tumor cells. Following neutron irradiation, fission reaction occurs neutrons are captured by ^10^B. This results in high linear energy transfer (LET) alpha particles and recoiling lithium-7 nuclei. Since these high-LET particles have limited path lengths (<10 μm) corresponding to one cell’s diameter, the lethal effect of these particles is confined to the ^10^B-containing cells [3]. Hence, the biological effects of the ^10^B (n,α) ^7^Li capture reaction depends on the concentration of ^10^B present in the tumor cells in the neutron-irradiated field with a concomitant sparing of normal cell.

Only two ^10^B drugs, boronophenylalanine (BPA) and sodium borocaptate (BSH) have been used clinically for BNCT and these were used separately or together in several clinical trials of BNCT against high-grade gliomas [1, 2]. At this time, BPA is a standard therapeutic drug in the clinical use of BNCT [2]. Since the L-amino acid transport system is highly expressed in tumor cells compared with normal cells in most organs including the brain, various boronated natural amino acids have been intensively tested. At this point, BPA is considered to be a better ^10^B delivery agent than BSH.

In general, inorganic ^10^B compounds as represented by BSH have less selectivity for tumors compared with BPA because of their lack of a cell-specific import system. Moreover, since BSH can penetrate into the brain only when the blood-brain barrier (BBB) is disrupted, BSH is poorly distributed to the tumor-infiltrating areas with an intact BBB. However, compared with BPA, BSH contains 12 times more ^10^B per molecule, which is another significant advantage of BSH over BPA.

**Fig. 1:**
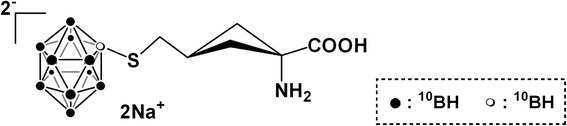
The chemical structure of ACBC-BSH

ACBC (1-amino-3**-**fluorocyclobutane-1-carboxylic acid) is an unnatural amino acid actively taken up by cells with L-type amino acid transporters [4]. Glioblastoma cells showed an intense uptake of ACBC [5], and fluoride-labeled ACBC ([^18^F]ACBC) was shown to be a useful tracer in positron emission tomography (PET) to detect malignant neoplasms [4, 6]. In the present study, we developed a novel BSH derivative conjugated with 1-amino-3**-**fluorocyclobutane-1-carboxylic acid: ACBC-BSH [7], the chemical structure of which is shown Fig. 1.

The purpose of the present study was 1] to determine the biodistribution of ACBC-BSH following either i.v. or i.t CED, and 2] to evaluate and compare it in vivo BNCT therapeutic efficiency compared to BPA using the F98 rat glioma model.

## Methods

### Boron compounds

BSH was purchased from Katchem (Prague, Czech Republic) and dissolved in sterile saline. The ACBC-BSH was prepared as previously described [7]. BPA (L-isomer) was kindly supplied by Stella Chemifa (Osaka, Japan) and was converted to a fructose complex [8].

### Cell culture

F98 rat glioma cells have been histologically characterized as an anaplastic astrocytoma. The F98 cells were a kind gift from Dr. Rolf Barth (Dept. of Pathology, The Ohio State University, Columbus, OH) and cultivated in Dulbecco’s Modified Eagle Medium (DMEM) supplemented with 10% fetal bovine serum and 10% penicillin/streptomycin/amphotericin B at 37 °C in a 5% CO_2_ atm. All of the materials for cell culture were purchased from Gibco Invitrogen (Grand Island, NY).

### The F98 Rat glioma model

All procedures were performed in accord with the guide for the care and use of laboratory animals as approved by the Animal Use Review Board and Ethical Committee of Osaka Medical College (No. 25092) and Kyoto University Research Reactor Institute (KUR; Kumatori, Osaka, Japan, No. 25030). Eight-week-old male Fischer 344 rats weighing 200–230 g were used (Japan SLC, Hamamatsu, Shizuoka). Each rat was anesthetized with an intraperitoneal injection of pentobarbital sodium (64.8 mg/kg), and the head was fixed with a stereotactic frame (Model 900; David Kopf Instruments, Tujunga, CA). The scalp was incised at the midline, and a small burr hole was made at the fixed target (1 mm posterior and 4 mm right lateral to the bregma) using an electric drill.

A 25-μL Hamilton microsyringe with a 26-ga. needle (model 1700RN, Hamilton Bonaduz, Bonaduz, Switzerland) was used for F98 tumor cell transplantation into the rat brain. The needle was first inserted to 6-mm depth from the skull and then withdrawn 1 mm to the target in the brain (5 mm from the skull). F98 cells diluted in 10 μL of DMEM at a concentration of either 10^3^ cells for the therapeutic experiments or 10^5^ cells for the biodistribution experiments were injected at a rate of 1 μL/min by an automatic infuser pump. The needle was kept in place for 1 min after the infusion and was then slowly withdrawn. The needle entry port in the burr hole was immediately sealed with bone wax, and the incised scalp was closed with a sterilized clip. This F98 brain tumor model rat is resistant to standard treatment with temozolomide and photon irradiation [9].

### The In vitro uptake of boron drugs in F98 cells

The intracellular ^10^B concentrations were measured by means of inductively coupled plasma atomic emission spectroscopy (ICP-AES). For the in vitro boron uptake studies, F98 rat glioma cells were used. First, 10^5^ F98 glioma cells were seeded into a tissue culture dish (100 × 20 mm; Becton Dickinson, Franklin Lakes, NJ) with culture medium (described above) at 37 °C in a 5% CO2 atm. After incubation for 5 days at 37 °C, the medium was replaced with culture medium (described above) containing 1 mM of ACBC-BSH, BSH, or BPA, and the cells were incubated for an additional 24 h at 37 °C. The medium then was removed, and the cells were washed twice with phosphate-buffered saline (PBS) and detached with trypsin-ethylenediamine tetraacetic acid solution. PBS was then added, and the cells were centrifuged twice and counted and sedimented.

The cells were then digested for 1 week with 1 N nitric acid solution (Wako Pure Chemical Industries, Osaka, Japan), and the boron uptake was determined by inductively coupled plasma atomic emission spectroscopy (ICP-AES: Hitachi, Tokyo). Pairwise comparisons were conducted using Student’s *t*-test. Group differences resulting in *p*-values < 0.05 were considered significant.

### Implantation of the Alzet osmotic pump for convection-enhanced delivery

For the convection-enhanced delivery (CED) of the three ^10^B compounds, the Alzet osmotic pump (model #2001D, Durect, Cupertino, CA) and the brain infusion kits consisting of a 28-ga., 5-mm-long rigid stainless steel cannula (Durect) were assembled and filled with 200 μL of the boron compound solution. After the glioma-bearing rat was anesthetized, the pump was implanted subcutaneously. A needle connecting to the infusion cannula was inserted via the same burr hole that had been made for the tumor implantation. The tip of the needle was placed 5 mm below the skull. A total of 1.2 mg ^10^B/kg rat body weight (b.w.) of BPA, BSH or ACBC-BSH was delivered at a constant flow rate of 8 μL/h for 24 h.

### The in vivo Biodistribution experiments

After the tumor implantation, when the rats showed signs suggesting that the tumor growth had become symptomatic (i.e., weight loss, lethargy, hunching, and ataxia), in vivo biodistribution studies were initiated. ACBC-BSH, BSH or BPA was administered intravenously to glioma-bearing rats at equivalent amounts of ^10^B adjusted to 12 mg ^10^B/kg b.w. ACBC-BSH, was administered i.t. by CED (1.2 mg ^10^B/kg b.w.) by means of Alzet osmotic pumps. The biodistribution of the test agents was determined at 1 and 6 h after the intravenous (i.v.) administration, and at 1, 6 and 24 h after the CED using 3 numbers of rats per group. The rats were euthanized, and the tumor, normal brain, blood, heart, lung, liver, spleen, kidney, skin and muscle were removed and weighed. The amount of ^10^B in each organ was quantified by ICP-AES. Pairwise comparisons were conducted using Student’s *t*-test. Group differences resulting in *p*-values <0.05 were considered significant.

### The intracellular distribution of each boron compound visualized by immunohistochemical staining

Immunostaining was performed to determine the incorporation of boron-containing amino acids into F98 cells, as described by Hattori et al. [7] with some modifications. We seeded glass coverslips coated with Matrigel (3.5 μg/cm^2^ protein) with F98 cells (0.8 × 10^5^ cells suspended in 3 mL of DMEM), and allowed these glass coverslips which be seeded with F98 cells to settle for 1 h at 37 °C. The medium was replaced with an equivalent amount of medium containing ACBC-BSH, BSH, or BPA (the final concentration was 1 mM in each case), and the cells were cultured for 24 h at 37 °C. Following washing with DMEM, the F98 cells were fixed with 10% paraformaldehyde in PBS for 10 min at room temperature and then rinsed with PBS and treated with 0.05% Triton X-100 for 10 min at room temperature.

Next, the cells were washed with PBS and preincubated in a humid chamber with 1.0% bovine serum albumin (BSA)/0.02% NaN3 in PBS at room temperature, followed by incubation with the anti-BSH monoclonal antibody A9H3 [10] for ACBC-BSH and BSH or the anti-BPA monoclonal antibody 2B10 [11] for BPA in PBS containing 1.0% BSA/0.02% NaN3 (0.2 μg/mL) for 60 min at 32 °C. The cells were rinsed with PBS and then incubated with Alexa-Fluor® 488Goat Anti-Mouse IgG in PBS containing BSA/NaN3 (described above) for 30 min at 32 °C. After being washed with PBS, the cells were mounted with Permafluor and then photographed with a microscope (IX-70, Olympus, Tokyo) equipped with a cooled charge-coupled device camera (UIC-QE; Molecular Devices, Sunnyvale, CA) controlled by MetaMorph software (Molecular Devices).

### In vivo therapy studies

Studies to evaluate the efficacy of BNCT were performed with the Kyoto University reactor (KUR), 14 days after the intracerebral implantation of F98 10^3^ glioma cells. Forty glioma bearing rats were randomly divided into five groups consisting of six animals each with an equal distribution of body weights. Group 1: untreated controls, Group 2: neutron irradiation only, Group 3: neutron irradiation following BPA/i.v., Group 4: neutron irradiation following ACBC-BSH/CED, Group 5: neutron irradiation following the combination of ACBC-BSH/CED and BPA/i.v. (the combined group).

For the in vivo BNCT studies, ACBC-BSH or BPA was administered to F98 glioma-bearing rats by means of CED or i.v., respectively. After the animals were anesthetized with sodium pentobarbital, all of their bodies, (except for their head), were shielded with ^6^LiF ceramic tiles in order to reduce the whole-body exposure. At 1 h after the termination of CED or the i.v. administration of the boron compound, the rats were irradiated at a reactor power of 1 MW with the Heavy Water Neutron Irradiation Facility (HWNIF) for 60 min.

The therapeutic effects of BNCT were evaluated by measuring the rats’ survival times. The mean survival time (MST), standard deviation (SD), and median survival time (MeST) were calculated and Kaplan-Meier survival curves were plotted for all groups. An overall log rank test was performed to test the equality of the survival curves over the groups.

### Estimation of the physical dose and biologically photon equivalent dose delivered to F98 glioma-bearing rats in the BNCT experiments

For the radiation dose analysis of the BNCT experiments, we estimated the physical dose and the biologically photon equivalent dose delivered to the rats as described below. The BNCT dose is the sum total of the physical dose attributed to the ^10^B(n,α)^7^Li, ^14^N(n,p)^14^C, and ^1^H(n,n)^1^H capture reactions and γ-ray. It is described by using the following equation:

$$ \mathrm{Physical}\ \mathrm{dose}\left(\mathrm{Gy}\right)={\mathrm{D}}_{\mathrm{B}}+{\mathrm{D}}_{\mathrm{N}}+{\mathrm{D}}_{\mathrm{H}}+\mathrm{D}\gamma $$

**Fig. 2:**
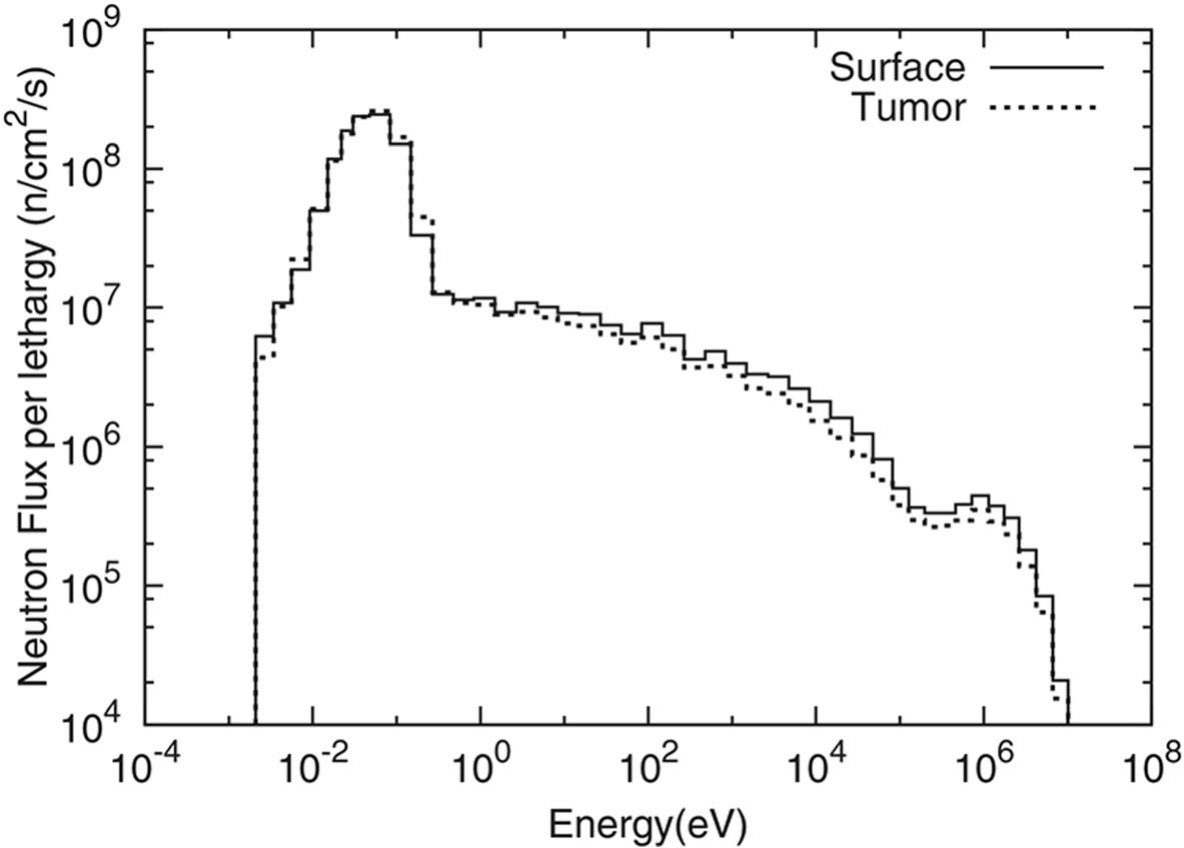
The neutron spectrum at the surface corresponding to the position of gold foils and tumor site

Here, D_B_ is the physical dose of boron derived from the equation of 7.43 × 10^−14^ (Gy cm^2^/μg ^10^B/g) × boron concentration (μg ^10^B/g) × thermal neutron fluence (1/cm^2^). D_N_ is the physical dose of nitrogen derived from the equation of 6.78 × 10^−14^ (Gy cm^2^/wt.%) × nitrogen concentration (weight %) × thermal neutron fluence (1/cm^2^). The numerical values above were taken from a previous study [12]. The physical dose of boron and nitrogen is caused by a capture reaction between the thermal neutrons and each nucleus. The thermal neutron fluence was measured by the radioactivity of gold foil (0.05 mm thick, 3 mm dia.) that was set at the surface of the rat head. D_H_ is the physical dose of hydrogen that is caused by the elastic scattering between epithermal or fast neutrons and the hydrogen nucleus. The simulation using a general Monte Carlo N-particle transport code [13] was performed in order to correct all dose component caused by the difference of neutron spectrum between the surface and tumor site. The simulation was performed with the following assumption of the dimension of the rat head. The diameter of the rat head was 25 mm. The tumor with the diameter of 5 mm was set in the rat head with the 5 mm distance from the surface. Figure 2 shows the neutron spectrum at the surface corresponding to the position of gold foils and tumor site. According to the results of the simulation, thermal neutron flux at the tumor site was 2% smaller than at the surface of the rat head. On the other hand, the epithermal and fast neutron flux at tumor site was 30% smaller than that at the surface of the rat head. Therefore, in the evaluation of tumor and brain dose, the physical doses of D_B_ and D_N_ were corrected to be 2% lower than these doses at the surface. Furthermore, D_H_ was corrected to be 30% lower than hydrogen dose evaluated by the surface neutron spectrum which was normalized by the measured thermal neutron fluence. Finally, the γ-ray dose was corrected to be 25% larger than the γ-ray dose measured by a thermoluminescence dosimeter at the surface of the rat head.

The biologically equivalent total photon dose, hereafter referred to as the equivalent dose in BNCT was estimated using the following equation.

$$ \mathrm{Equivalent}\ \mathrm{dose}\ \left(\mathrm{Gy}-\mathrm{E}\mathrm{q}\right)={\mathrm{D}}_{\mathrm{B}}\times {\mathrm{CBE}}_{\mathrm{B}}+{\mathrm{D}}_{\mathrm{N}}\times {\mathrm{RBE}}_{\mathrm{N}}+{\mathrm{D}}_{\mathrm{H}}\times {\mathrm{RBE}}_{\mathrm{H}}+\mathrm{D}\gamma \left(\mathrm{Gy}\right) $$

CBEB is the compound biological effectiveness (CBE) for boron compounds, and in the case of BPA, this value is 3.8 [14] for tumor tissue and 0.9 [15] for the normal brain. The relative biological effectiveness (RBE) for nitrogen (RBEN) is 3.0. The RBE for hydrogen (RBEH) is also 3.0 [16]. In the present study, we used 0.9 (this value was calculated according to a previously described [15]) as CBE value for the normal brain because ^10^B concentration ratio of the normal tissue to the blood (N/B) of BPA-i.v.-1 h was 3.0/8.5 = 0.35. On the other hand, in our clinical study [1, 2], we used 1.35 as CBE value for the normal brain because N/B was 0.63.

The physical dose for ACBC-BSH can be evaluated, but we were unable to estimate the equivalent dose because the CBE_B_ for ACBC-BSH is unknown. The nitrogen concentration was estimated as 2.6% based on International Commission on Radiation Units and Measurement (ICRU) four tissue components.

## Results

### The In vitro uptake of boron drugs in F98 glioma cells

**Fig. 3:**
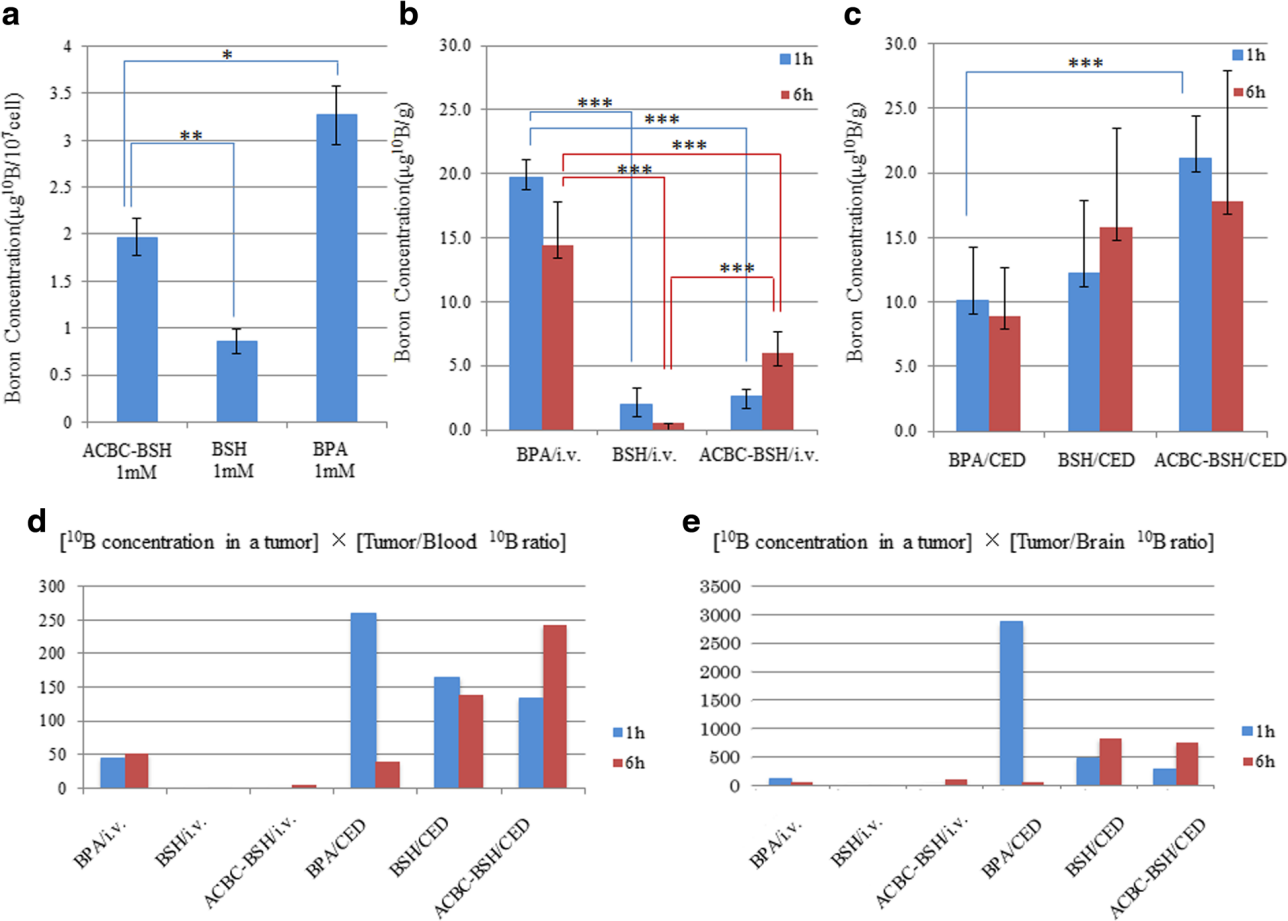
**a** Boron concentration of F98 glioma 24 h after incubation in media including 1 mM of each boron compounds. The boron concentrations of tumor cells were significantly higher in the ACBC-BSH than in the BSH (1.97 ± 0.2 μg B/10^7^ cells and 0.86 ± 0.1 μg B/10^7^ cells). The boron concentrations of tumor cells were the highest in the BPA. *, *P* = 0.033; **, *P* = 0.0014. **b** Boron concentrations in a tumor in F98 glioma bearing rats, when we administrated BPA, BSH, and ACBC-BSH at the dose of 12 mg ^10^B/kg by i.v. ***, *P* < 0.05. **c** Boron concentrations in a tumor in F98 glioma bearing rats, when we administrated BPA, BSH, and ACBC-BSH at the dose of 1.2 mg ^10^B/kg by CED. ***, *P* < 0.05. **d** Product values of the intratumoral ^10^B concentration and the tumor/normal tissues ^10^B ratio; [^10^B concentration in a tumor] x [Tumor/Blood ^10^B ratio]. **e** Product values of the intratumoral ^10^B concentration and the tumor/normal tissues ^10^B ratio; [^10^B concentration in a tumor] x [Tumor/Brain ^10^B ratio]

Intracellular ^10^B concentrations were measured by means of ICP. The ^10^B concentrations in F98 glioma cells at 24 h after their exposure to 1 mM of each ^10^B drug are summarized in (Fig. 3a). BPA showed the highest concentration (3.27 ± 0.3 μg ^10^B/10^7^ cells) compared with ACBC-BSH (1.97 ± 0.2 μg ^10^B/10^7^ cells; *p* = 0.0038) and BSH (0.86 ± 0.1 μg ^10^B/10^7^ cells; *p* = 0.0003). ACBC-BSH also showed a significantly higher B^10^ concentration than BSH (*p* = 0.0014).

### In vivo Biodistribution experiments

We measured the ^10^B concentrations in tumor, normal brain and blood in F98 glioma-bearing rats after the administration of BPA, BSH, and ACBC-BSH at the dose of 12 mg ^10^B/kg by the i.v. route or 1.2 mg ^10^B/kg by CED and the data are summarized in Table 1 and the intratumoral (i.t.) ^10^B localization of each ^10^B drug are shown in Fig. 3b and c.

**Table 1:** Summary of Boron biodistribution in F98 glioma bearing rats

				Boron concentrations ± SD(μg^10^B/g)									Ratios^b^	
Agent	Route	Time^a^(h)	n	Blood			Brain			Tumor			T/Bl	T/Br
ACBC-BSH	CED^c^ ^200μl/24h^	1	3	3.3	±	2.2	1.5	±	1.3	21.1	±	3.3	6.4	14.2
		6	3	1.3	±	0.0	0.4	±	0.3	17.8	±	10.1	13.7	42.4
		24	3	0.5	±	0.1	0.0	±	0.0	0.0	±	0.1	0.1	1.4
	i.v.^d^	1	4	6.3	±	1.7	0.2	±	0.0	2.7	±	0.5	0.4	14.9
		6	3	7.0	±	3.4	0.3	±	0.1	6.0	±	1.7	0.9	18.6
BPA	CED^e^ ^200μl/24h^	1	3	0.5	±	0.3	0.1	±	0.1	10.1	±	4.1	23	125.2
		6	2	2.0	±	0.9	0.7	±	0.1	8.9	±	3.7	4.4	13.7
		24	3	0.4	±	0.3	0.0	±	0.0	0.1	±	0.2	0.4	7
	i.v.^f^	1	3	8.5	±	0.3	3.0	±	0.6	19.7	±	1.4	2.3	6.7
		6	3	4.1	±	1.3	3.1	±	1.3	14.4	±	3.4	3.6	4.7
BSH	CED^g^ ^200μl/24h^	1	3	0.9	±	0.7	0.3	±	0.2	12.2	±	5.6	13.6	40.7
		6	3	1.8	±	0.5	0.3	±	0.5	15.8	±	7.7	8.8	52.7
		24	3	1.6	±	0.3	0.1	±	0.0	1.4	±	0.3	0.9	14.0
	i.v.^h^	1	3	10.3	±	3.3	0.1	±	0.1	2.0	±	1.3	0.2	20.0
		6	3	2.0	±	0.3	0.1	±	0.0	0.5	±	0.0	0.3	5.0

*ACBC* α-aminocyclobutane-1-carboxylic acid, *CED* convection-enhanced delivery, *SD* standard deviation, *BPA* p-boronphenylalanine, *BSH* dodecaboranethiol, *iv* intravenous

^a^Time to euthanize rats after boron compound administration

^b^T/Bl indicates the tumor to blood ratio and T/Br indicates the tumor to normal brain ratio

^c^ACBC-BSH was administered by long term CED in volume of 200 μl for 24 h at a dose of 1.2 mg^10^B/kg b.w

^d^ACBC-BSH was administered i.v. at a dose of 12 mg10B/kg b.w

^e^BPA was administered by long term CED in volume of 200 μl for 24 h at a dose of 1.2 mg^10^B/kg b.w

^f^BPA was administered i.v. at a dose of 12 mg^10^B/kg b.w (= 250 mgBPA/kg b.w)

^g^BSH was administered by long term CED in volume of 200 μl for 24 h at a dose of 1.2 mg^10^B/kg b.wl

^h^BSH was administered i.v. at a dose of 12 mg^10^B/kg b.w

In the i.v. administration experiment (Fig. 3b), BPA showed significantly higher ^10^B concentrations in the tumors among the three drugs used, at both 1 h (BPA 19.7 ± 1.4 vs. BSH 2.0 ± 1.3 μg ^10^B/g, *p* = 0.0001; vs. ACBC-BSH 2.7 ± 0.5 μg ^10^B/g, *p* = 0.000003) and 6 h (BPA 14.4 ± 3.4 vs. BSH 0.5 ± 0.0 μg ^10^B/g, *p* = 0.0189; vs. ACBC-BSH 6.0 ± 1.7 μg ^10^B/g, *p* = 0.0180) after the injection. ACBC-BSH was also significantly and highly accumulated in the tumors compared with BSH at 6 h post-injection (6.0 ± 1.7 vs. 0.5 ± 0.0 μg ^10^B/g, *p* = 0.0299).

CED of (Fig. 3c), ACBC-BSH resulted in significantly higher i.t. ^10^B concentrations compared with those of BPA and BSH at 1 h (ACBC-BSH 21.1 ± 3.3 vs. BPA 10.1 ± 4.1 μg ^10^B/g, *p* = 0.0225; vs. BSH 12.2 ± 5.6 μg ^10^B/g, *p* = 0.0753), respectively. However, at 6 h (ACBC-BSH 17.8 ± 10.1 vs. BPA 8.9 ± 3.7 μg ^10^B/g, *p* = 0.2263; vs. BSH 15.8 ± 7.7 μg ^10^B/g, *p* = 0.8018) this was not the case. In contrast, BSH did not show any significant difference compared with BPA at 1 h (BSH 12.2 ± 5.6 vs. BPA 10.1 ± 4.1 μg ^10^B/g, *p* = 0.6301) or at 6 h (BSH 15.8 ± 7.7 vs. BPA 8.9 ± 3.7 μg ^10^B/g, *p* = 0.2353) post-CED.

The i.t. ^10^B concentration and the tumor/normal tissues^10^B ratio: [^10^B concentration in a tumor] × [Tumor/Blood ^10^B ratio] and [^10^B concentration in a tumor] × [Tumor/Brain ^10^B ratio] are as shown in Fig. 3d, e. The i.t. ^10^B concentration is a main factor correlated with the therapeutic efficacy of BNCT in the suppression of tumor growth. On the other hand, the tumor/normal tissue ratio of a ^10^B concentration is a key factor for the safety of the normal cells that are included in a neutron-irradiated field. Since both of these factors are important to ensure the quality of BNCT, we calculated their product values as indexes reflecting a favorable biodistribution of each ^10^B drug.

The i.t. ^10^B concentration of each drug was similar between the i.v. (12 mg ^10^B/kg) and CED (1.2 mg ^10^B/kg), but these indexes, i.e., the [^10^B concentration in a tumor] × [Tumor/Blood ^10^B ratio] and [^10^B concentration in a tumor] × [Tumor/Brain ^10^B ratio] were much higher following CED compared with i.v. administration for all three drugs (Fig. 3d, e). The boron concentrations in the liver, spleen, kidney, heart, lung, muscle, and skin following CED were < 4.4 μg ^10^B/g, which were much lower compared with i.v. administration (data not shown).

### Intracellular distribution of each boron compound visualized by immunochemical staining

**Fig. 4:**
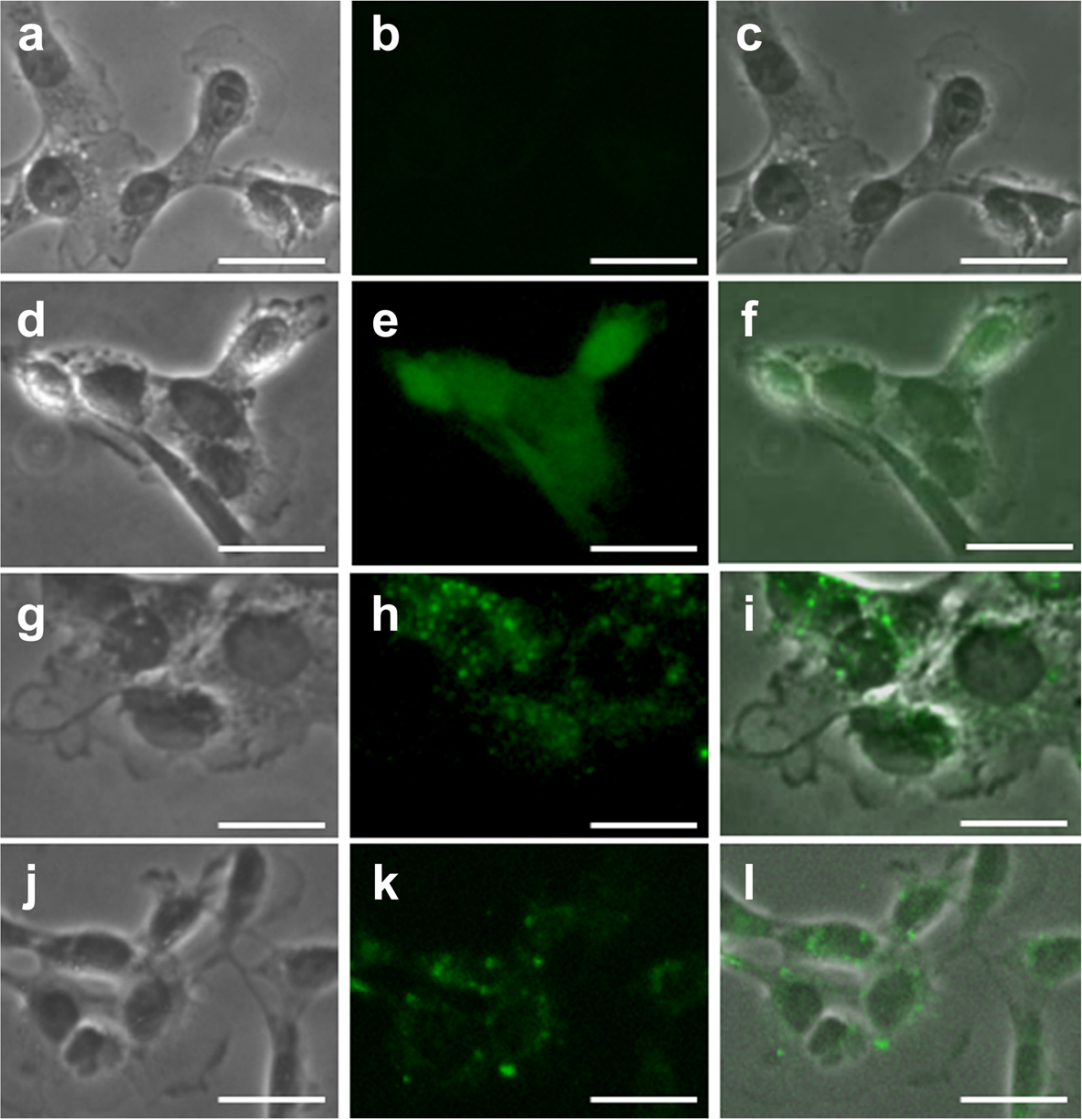
The microdistribution of ACBC-BSH and BPA and BSH in F98 cells. Scale bar =20 μm. **a** A phase-contrast micrograph of F98 cells that were cultured in DMEM. **b** A fluorescence micrograph of F98 cells that were cultured in DMEM stained with the anti-BSH antibody A9H3. **c** A merged image of A, B. **d** A phase-contrast micrograph of F98 cells that were cultured in DMEM containing BPA. **e** A fluorescence micrograph of F98 cells that were cultured in DMEM containing BPA stained with the anti-BPA antibody 2B10. **f** A merged image of D, E. **g** A phase-contrast micrograph of F98 cells that were cultured in DMEM containing ACBC-BSH. **h** A fluorescence micrograph of F98 cells that were cultured in DMEM containing ACBC-BSH stained with the anti-BSH antibody A9H3. **i** A merged image of G, H. **j** A phase-contrast micrograph of F98 cells that were cultured in DMEM containing BSH. **k** A fluorescence micrograph of F98 cells that were cultured in DMEM containing BSH stained with the anti-BSH antibody A9H3. **l** A merged image of J, K

Figure 4 showed immunohistochemical staining. The immunohistochemical staining showed that the distributions of ACBC-BSH and BSH were different from that of BPA. BPA was distributed homogeneously over the cell nucleus and cytoplasm (Fig. 4d, e, f). In contrast, ACBC-BSH and BSH were incorporated into the cell membrane of the F98 cells and aggregated on the fringe of the cell nuclei (Fig. 4g, h, i, j, k, l).

### In vivo therapeutic experiments

We performed therapeutic BNCT experiments using five groups of rats, and the MST of each group was as follows: untreated controls, 27.2 ± 2.4 days; neutron-irradiation controls, 29.8 ± 1.9 days; BNCT with BPA/i.v., 37.4 ± 2.6 days; BNCT with ACBC-BSH/CED, 37.0 ± 5.2 days, and BNCT concomitantly using BPA/i.v. and ACBC-BSH/CED (the combined treatment group), 44.3 ± 8.0 days.

**Fig. 5:**
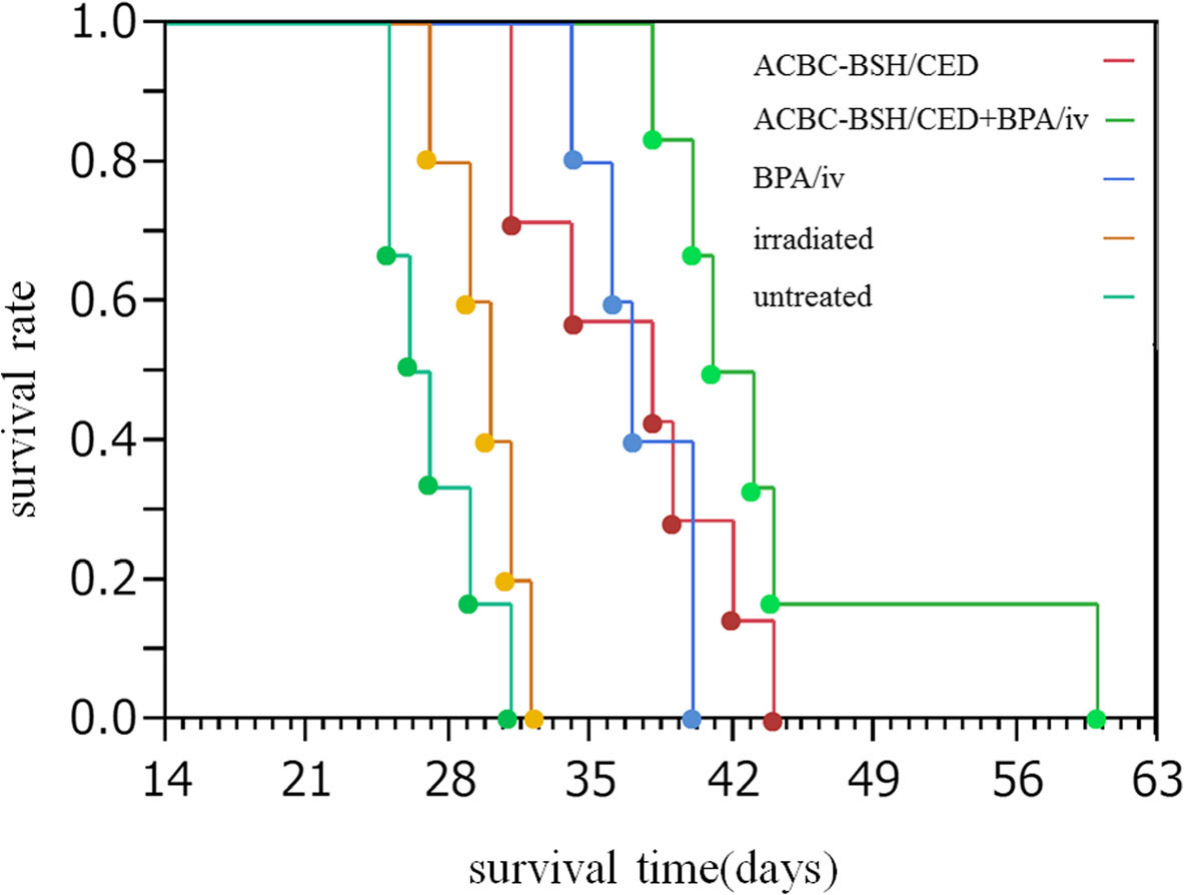
Kaplan-Meier survival curves for F98 glioma cells bearing rats following CED delivery of ACBC-BSH and/or i.v. injection of BPA followed by BNCT. Survival times in days after implantation have been plotted for untreated animals, irradiated controls, BPA/i.v., ACBC-BSH/CED, ACBC-BSH/CED + BPA/i.v. groups (*n* = 3 each)

A summary of the boron concentrations and physical and equivalent doses delivered to F98 glioma-bearing rats is given in Table 2. The highest physical dose delivered to the tumor was 3.7 Gy in the ACBC-BSH/CED group, and the corresponding dose to the normal brain was 1.1 Gy. The survival data following BNCT are summarized in Table 3, and the Kaplan-Meier survival plots are shown in Fig. 5.

**Table 2:** Summary of Boron concentrations and Physical doses and Equivalent doses delivered in F98 glioma bearing rats

			Boron concentrations ± SD(μg10B/g)						Physical dose^b^ (Gy)		Equivalent dose^c^ (Gy-Eq)	
Agent	Route	Time^a^(h)	Brain			Tumor			Brain	Tumor	Brain	Tumor
ACBC-BSH	CED	1	1.5	±	1.3	21.1	±	3.3	1.1	3.7	-	-
BPA	iv	1	3.0	±	0.6	19.7	±	1.4	1.3	3.5	2.2	11.7
Irradiated Controls	-	-	0	±	0	0	±	0	0.9	0.9	-	-
Untreated Controls	-	-	0	±	0	0	±	0	0	0	0	0

*D*_*B*_ Boron dose, *D*_*N*_ neutron dose, *D*_*H*_ hydrogen dose, *D*_*γ*_ gamm-ray dose

CBE_B_ is from CBE(Compound Biological Effectiveness) for D_B_ and, in the case of BPA, this value is 3.8 for the tumor tissue and 0.9 for the normal brain

RBE_N_ is from RBE(Relative Biological Effectiveness) for D_N_ and this value is 3.0

RBE_H_ is from RBE(Relative Biological Effectiveness) for D_H_ and this value is 3.0

^a^Time to euthanize rats after boron compound administration

^b^Physical dose estimates include contributions from gamma photons,10B(n,α)7Li, 14 N(n,p)14C, and 1H(n,n)1H reactions

^c^Equivalent dose is a value calculated using the D_B_ x CBE_B_ + D_N_ x RBE_N_ + D_H_ x RBE_H_ + D_γ_

**Table 3:** Survival times of F98 glioma bearing rats following i.c. delivery of BPA and ACBC-BSH by iv or CED

Agent/Route		Survival Time					%ILS^b^
Group	n^a^	Mean ± SD			Median	Range	Median
ACBC-BSH/CED + BPA/iv	6	44.3	±	8.0	42.0	38–60	59.2
ACBC-BSH/CED	7	37.0	±	5.2	38.0	31–44	36.0
BPA/iv	5	37.4	±	2.6	37.0	34–40	37.5
Irradiated Controls	5	29.8	±	1.9	30.0	27–32	9.6
Untreated Controls	6	27.2	±	2.4	26.5	25–31	-

*ACBC* α-aminocyclobutane-1-carboxylic acid, *CED* convection-enhanced delivery, *SD* standard deviation, *BPA* p-boronphenylalanine, *BSH* dodecaboranethiol, *iv* intravenous

^a^n is the number of animals per group

^b^Percent increace life span(%ILS) was defined relative to the mean survival times of untreated controls

As shown in Fig. 5, the MST in all BNCT groups (i.e., the BNCT with BPA/i.v. group, the BNCT with ACBC-BSH/CED group, and the combined treatment group) showed significantly longer survival compared with the neutron-irradiation control group (*p* = 0.005, *p* = 0.002, and *p* = 0.0007, respectively). In addition, the combined treatment group showed significantly longer MSTs compared with the BNCT with BPA/i.v. group (*p* = 0.0382) and the highest percent increased lifespan (%ILS) value among all treated groups (59.2%; Table 3).

## Discussion

Extensive studies relating to the use of unnatural amino acids as boron delivery agents for BNCT have been reported by Barth, Chandra and Kabalka [17–19]. In the present study, we designed and synthesized ACBC-BSH, which is chemically modified BSH with a tumor-avid synthetic unnatural amino acid, ACBC. BSH is a stable polyhedral borane that has been shown to be safe in clinical trials of BNCT, although it does not penetrate into normal brain with an intact blood-brain barrier (BBB). On the other hand, intravenously administered BPA penetrates the BBB by L-amino acid transporters. As reported by Barth et al although intravenously administered BPA may penetrate the BBB, it has been shown that intracarotid (i.c.) administration, combined with BBB disruption, significantly enhanced uptake of BPA in F98 glioma bearing rats [20, 21]. Compared with BPA, BSH contains 12 times more ^10^B atoms per molecule, which is a significant advantage of BSH over BPA. It has been reported that ACBC could be transported into cells by L-amino acid transporters like phenylalanine and BPA [22, 23], and the PET using F18-labeled ACBC revealed its tumor selective accumulation [6]. Thus, it may also be useful to visualize and quantify the distribution of ACBC-BSH in patients with malignant brain tumors, using PET.

PET is also useful for the detection of brain tumors [24, 25]. If [^18^F]ACBC-PET can be used in clinical practice, it may become a useful modality for deciding whether to use BNCT with ACBC-BSH. Therefore, in the present study, we designed and synthesized ACBC-BSH and elucidated its biodistribution and therapeutic efficacy in BNCT against malignant glioma. We observed that it was difficult for ACBC-BSH to pass through the BBB because of its higher molecular weight. We adopted CED for the drug administration to solve this problem.

CED is an innovative method for local drug delivery to brain tumors, by which a pressure gradient, or bulk flow, is used to drive an infusate through the extracellular fluid compartment. As demonstrated in animal studies [26–30], CED not only increased the delivery of both low- and high-molecular-weight agents, but also improved their therapeutic efficacy. Indeed, we succeeded in achieving a high accumulation of boron in the tumors of rats in which ACBC-BSH was administered by CED, compared with ACBC-BSH administered intravenously. These results suggest that CED can deliver a variety of therapeutic agents to tumor-infiltrating areas where the BBB seems to be intact. In the present study, it was demonstrated that ACBC-BSH accumulated peripherally on cell nuclei. In vivo, rats that received ACBC-BSH by CED had higher [^10^B concentration in a tumor] × [Tumor/Blood ^10^B ratio] (1 and 6 h) and [^10^B concentration in a tumor] × [Tumor/Brain ^10^B ratio] (1 and 6 h) values compared with BPA/i.v., although the total amount of ^10^B of the ACBC-BSH administered by CED rats (1.2 mg ^10^B/kg b.w.) was one-tenth that of BPA/i.v. (12 mg^10^B/kg b.w.).

This would suggest that ACBC-BSH administered by CED would be expected to have a greater therapeutic than BPA administered by i.v.. However, since higher advantage index includes the score for safety and also ^10^B concentrations, these were almost equivalent among the two treatment groups following neutron irradiation, the superiority of BNCT using ACBC-BSH/CED was not detected. However, if higher neutron dose was used to irradiate the tumors, it might be more effective. With CED of ACBC-BSH the ^10^B atoms would be expected to localize at along the periphery of the cell membrane. The dyshomogeneous subcellular distribution within cells would reduce its therapeutic effecting. However, BPA has a more homogeneous distribution within the cell and it would be advantageous to use both of these compounds in combination.

## Conclusions

Our study demonstrated that the combined administration of ACBC-BSH/CED and BPA/i.v. resulted a modest but statistically significant increase in the MST of F98 glioma bearing rats compared with BPA/i.v. suggesting that further studies are warranted to using ACBC-BSH/CED as a boron delivery agent.

## Abbreviations

%ILS: Percent increased lifespan
^10^B: Boron-10
ACBC: 1-amino-3**-**fluorocyclobutane-1-carboxylic acid
b.w.: Body weight
BBB: Blood-brain barrier
BNCT: Boron neutron capture therapy
BPA: Boronophenylalanine
BSA: Bovine serum albumin
BSH: Sodium borocaptate
CBE: Compound biological effectiveness
CED: Convection-enhanced delivery
DMEM: Dulbecco’s Modified Eagle Medium
HWNIF: Heavy water neutron irradiation facility
i.c.: intracarotid
i.t.: intratumoral
i.v.: intravenous
ICP-AES: Inductively coupled plasma atomic emission spectroscopy
ICRU: International Commission on Radiation Units and Measurement
KUR: Kyoto University Research Reactor Institute
LET: Linear energy transfer
MeST: Median survival time
MST: Mean survival time
PBS: Phosphate-buffered saline
PET: Positron emission tomography
RBE: Relative biological effectiveness
SD: Standard deviation

## References

[1] Kawabata S, Miyatake S, Kuroiwa T, Yokoyama K, Doi A, Iida K, Miyata S, Nonoguchi N, Michiue H, Takahashi M (2009). Boron neutron capture therapy for newly diagnosed glioblastoma. J Radiat Res.

[2] Kawabata S, Miyatake S, Hiramatsu R, Hirota Y, Miyata S, Takekita Y, Kuroiwa T, Kirihata M, Sakurai Y, Maruhashi A, Ono K (2011). Phase II clinical study of boron neutron capture therapy combined with X-ray radiotherapy/temozolomide in patients with newly diagnosed glioblastoma multiforme--study design and current status report. Appl Radiat Isot.

[3] Barth RF, Vicente MG, Harling OK, Kiger WS, Riley KJ, Binns PJ, Wagner FM, Suzuki M, Aihara T, Kato I, Kawabata S (2012). Current status of boron neutron capture therapy of high grade gliomas and recurrent head and neck cancer. Radiat Oncol.

[4] Rice SL, Roney CA, Daumar P, Lewis JS (2011). The next generation of positron emission tomography radiopharmaceuticals in oncology. Semin Nucl Med.

[5] Kimler BF (1994). The 9 L rat brain tumor model for pre-clinical investigation of radiation-chemotherapy interactions. J Neuro-Oncol.

[6] Shoup TM, Olson J, Hoffman JM, Votaw J, Eshima D, Eshima L, Camp VM, Stabin M, Votaw D, Goodman MM (1999). Synthesis and evaluation of [18 F]1-amino-3-fluorocyclobutane-1-carboxylic acid to image brain tumors. J Nucl Med.

[7] Hattori Y, Kusaka S, Mukumoto M, Ishimura M, Ohta Y, Takenaka H, Uehara K, Asano T, Suzuki M, Masunaga SI, et al. Synthesis and in vitro evaluation of thiododecaborated alpha, alpha- cycloalkylamino acids for the treatment of malignant brain tumors by boron neutron capture therapy. Amino Acids. 2014. 10.1007/s00726-014-1829-5 25173737

[8] Coderre JA, Button TM, Micca PL, Fisher CD, Nawrocky MM, Liu HB (1994). Neutron capture therapy of the 9 L rat gliosarcoma using the p-boronophenylalanine-fructose complex. Int J Radiat Oncol Biol Phys.

[9] Yang W, Huo T, Barth RF, Gupta N, Weldon M, Grecula JC, Ross BD, Hoff BA, Chou TC, Rousseau J, Elleaume H (2011). Convection enhanced delivery of carboplatin in combination with radiotherapy for the treatment of brain tumors. J Neuro-Oncol.

[10] Kirihata M, Asano T, Uehara K. Preparation of borocaptate(^10^B) (BSH) derivatives as hapten compound for production of antibody against BSH. PCT Int Appl. 2007. WO2007097065 A1 20070830. http://www.google.com/patents/US8865874.

[11] Kirihata M, Asano T. Phenylboronic acid derivative, antibodies to its conjugates with polymers, hybridomas producing the antibodies, determination of p-boronophenylalanine using the antibodies, and kits containing the antibodies. Jpn Kokai Tokkyo Koho. 2008. JP2008094729 A 20080424. http://www.ekouhou.net/%E3%83%8F%E3%83%97%E3%83%86%E3%83%B3%E5%8C%96%E5%90%88%E7%89%A9%E3%81%8A%E3%82%88%E3%81%B3%E6%8A%97%E4%BD%93/disp-A,2008-94729.html.

[12] Yamatomo N, Iwagami T, Kato I, Masunaga S, Sakurai Y, Iwai S, Nakazawa M, Ono K, Yura Y (2013). Sonoporation as an enhancing method for boron neutron capture therapy for squamous cell carcinomas. Radiat Oncol.

[13] Hughes HG, Brown FB, Bull JS, Goorley JT, Little RC, Liu LC, Mashnik SG, Prael RE, Selcow EC, Sierk AJ (2005). MCNP5 for proton radiography. Radiat Prot Dosim.

[14] Coderre JA, Makar MS, Micca PL, Nawrocky MM, Liu HB, Joel DD, Slatkin DN, Amols HI (1993). Derivations of relative biological effectiveness for the high-let radiations produced during boron neutron capture irradiations of the 9 L rat gliosarcoma in vitro and in vivo. Int J Radiat Oncol Biol Phys.

[15] Ono K (2016). An analysis of the structure of the compound biological effectiveness factor. J Radiat Res.

[16] Suzuki M, Kato I, Aihara T, Hiratsuka J, Yoshimura K, Niimi M, Kimura Y, Ariyoshi Y, Haginomori S, Sakurai Y (2014). Boron neutron capture therapy outcomes for advanced or recurrent head and neck cancer. J Radiat Res.

[17] Barth RF, Kabalka GW, Yang W, Huo T, Nakkula RJ, Shaikh AL, Haider SA, Chandra S (2014). Evaluation of unnatural cyclic amino acids as boron delivery agents for treatment of melanomas and gliomas. Appl Radiat Isot.

[18] Chandra S, Ahmad T, Barth RF, Kabalka GW (2014). Quantitative evaluation of boron neutron capture therapy (BNCT) drugs for boron delivery and retention at subcellular-scale resolution in human glioblastoma cells with imaging secondary ion mass spectrometry (SIMS). J Microsc.

[19] Kabalka GW, Shaikh AL, Barth RF, Huo T, Yang W, Gordnier PM, Chandra S (2011). Boronated unnatural cyclic amino acids as potential delivery agents for neutron capture therapy. Appl Radiat Isot.

[20] Barth RF, Yang W, Rotaru JH, Moeschberger ML, Joel DD, Nawrocky MM, Goodman JH, Soloway AH (1997). Boron neutron capture therapy of brain tumors: enhanced survival following intracarotid injection of either sodium borocaptate or boronophenylalanine with or without blood-brain barrier disruption. Cancer Res.

[21] Barth RF, Yang W, Rotaru JH, Moeschberger ML, Boesel CP, Soloway AH, Joel DD, Nawrocky MM, Ono K, Goodman JH (2000). Boron neutron capture therapy of brain tumors: enhanced survival and cure following blood-brain barrier disruption and intracarotid injection of sodium borocaptate and boronophenylalanine. Int J Radiat Oncol Biol Phys.

[22] Washburn LC, Sun TT, Anon JB, Hayes RL (1978). Effect of structure on tumor specificity of alicyclic alpha-amino acids. Cancer Res.

[23] Washburn LC, Sun TT, Byrd B, Hayes RL, Butler TA (1979). 1-aminocyclobutane[11C]carboxylic acid, a potential tumor-seeking agent. J Nucl Med.

[24] Ceyssens S, Van Laere K, de Groot T, Goffin J, Bormans G, Mortelmans L (2006). [11C]methionine PET, histopathology, and survival in primary brain tumors and recurrence. AJNR Am J Neuroradiol.

[25] Tsuyuguchi N, Takami T, Sunada I, Iwai Y, Yamanaka K, Tanaka K, Nishikawa M, Ohata K, Torii K, Morino M (2004). Methionine positron emission tomography for differentiation of recurrent brain tumor and radiation necrosis after stereotactic radiosurgery--in malignant glioma. Ann Nucl Med.

[26] Kawabata S, Yang W, Barth RF, Wu G, Huo T, Binns PJ, Riley KJ, Ongayi O, Gottumukkala V, Vicente MG (2011). Convection enhanced delivery of carboranylporphyrins for neutron capture therapy of brain tumors. J Neuro-Oncol.

[27] Miyata S, Kawabata S, Hiramatsu R, Doi A, Ikeda N, Yamashita T, Kuroiwa T, Kasaoka S, Maruyama K, Miyatake S (2011). Computed tomography imaging of transferrin targeting liposomes encapsulating both boron and iodine contrast agents by convection-enhanced delivery to F98 rat glioma for boron neutron capture therapy. Neurosurgery.

[28] Yang W, Barth RF, Adams DM, Ciesielski MJ, Fenstermaker RA, Shukla S, Tjarks W, Caligiuri MA (2002). Convection-enhanced delivery of boronated epidermal growth factor for molecular targeting of EGF receptor-positive gliomas. Cancer Res.

[29] Mardor Y, Rahav O, Zauberman Y, Lidar Z, Ocherashvilli A, Daniels D, Roth Y, Maier SE, Orenstein A, Ram Z (2005). Convection-enhanced drug delivery: increased efficacy and magnetic resonance image monitoring. Cancer Res.

[30] Barth RF, Yang W, Huo T, Riley KJ, Binns PJ, Grecula JC, Gupta N, Rousseau J, Elleaume H (2011). Comparison of intracerebral delivery of carboplatin and photon irradiation with an optimized regimen for boron neutron capture therapy of the F98 rat glioma. Appl Radiat Isot.

